# Supplementary material for: Getting More Out of Biomedical Documents with GATE's Full Lifecycle Open Source Text Analytics
Source: PLoS Comput Biol. 2013 Feb 7;9(2):e1002854. doi: 10.1371/journal.pcbi.1002854 (PMC3567135; doi:10.1371/journal.pcbi.1002854)
Supplement: Dataset S1 — GATE software. Dataset S1 bundled with this paper contains a distribution of GATE (or see http://gate.ac.uk/download/). (TGZ) [file pcbi.1002854.s001.tgz › plos-gate/plugins/Tagger_MetaMap/doc/info/olteanu/interfaces/package-tree.html]

info.olteanu.interfaces Class Hierarchy


---


|  |  |  |
| --- | --- | --- |
| **Overview** \| **Package** \| Class \| Use \| **Tree** \| **Deprecated** \| **Index** \| **Help** | |  |
| **PREV**   **NEXT** | **FRAMES**    **NO FRAMES**     **All Classes** |  |


---


## Hierarchy For Package info.olteanu.interfaces

**Package Hierarchies:**: All Packages

---

## Interface Hierarchy

- info.olteanu.interfaces.**StringFilter**

---


|  |  |  |
| --- | --- | --- |
| **Overview** \| **Package** \| Class \| Use \| **Tree** \| **Deprecated** \| **Index** \| **Help** | |  |
| **PREV**   **NEXT** | **FRAMES**    **NO FRAMES**     **All Classes** |  |


---
